# Tumor Infiltration in Enhancing and Non-Enhancing Parts of Glioblastoma: A Correlation with Histopathology

**DOI:** 10.1371/journal.pone.0169292

**Published:** 2017-01-19

**Authors:** Oliver Eidel, Sina Burth, Jan-Oliver Neumann, Pascal J. Kieslich, Felix Sahm, Christine Jungk, Philipp Kickingereder, Sebastian Bickelhaupt, Sibu Mundiyanapurath, Philipp Bäumer, Wolfgang Wick, Heinz-Peter Schlemmer, Karl Kiening, Andreas Unterberg, Martin Bendszus, Alexander Radbruch

**Affiliations:** 1 Department of Neuroradiology, University of Heidelberg Medical Center, Heidelberg, Germany; 2 Department of Radiology, German Cancer Research Center (DKFZ), Heidelberg, Germany; 3 German Cancer Consortium (DKTK), Radiology, Heidelberg, Germany; 4 Department of Neurosurgery, Division Stereotactic Neurosurgery, University of Heidelberg Medical Center, Heidelberg, Germany; 5 Department of Psychology, School of Social Sciences, University of Mannheim, Mannheim, Germany; 6 Department of Neuropathology, University of Heidelberg Medical Center, Heidelberg, Germany; 7 Department of Neurology, University of Heidelberg Medical Center, Heidelberg, Germany; Julius-Maximilians-Universität Würzburg, GERMANY

## Abstract

**Purpose:**

To correlate histopathologic findings from biopsy specimens with their corresponding location within enhancing areas, non-enhancing areas and necrotic areas on contrast enhanced T1-weighted MRI scans (cT1).

**Materials and Methods:**

In 37 patients with newly diagnosed glioblastoma who underwent stereotactic biopsy, we obtained a correlation of 561 1mm^3^ biopsy specimens with their corresponding position on the intraoperative cT1 image at 1.5 Tesla. Biopsy points were categorized as enhancing (CE), non-enhancing (NE) or necrotic (NEC) on cT1 and tissue samples were categorized as “viable tumor cells”, “blood” or “necrotic tissue (with or without cellular component)”. Cell counting was done semi-automatically.

**Results:**

NE had the highest content of tissue categorized as viable tumor cells (89% vs. 60% in CE and 30% NEC, respectively). Besides, the average cell density for NE (3764 ± 2893 cells/mm^2^) was comparable to CE (3506 ± 3116 cells/mm^2^), while NEC had a lower cell density with 2713 ± 3239 cells/mm^2^. If necrotic parts and bleeds were excluded, cell density in biopsies categorized as “viable tumor tissue” decreased from the center of the tumor (NEC, 5804 ± 3480 cells/mm^2^) to CE (4495 ± 3209 cells/mm^2^) and NE (4130 ± 2817 cells/mm^2^).

**Discussion:**

The appearance of a glioblastoma on a cT1 image (circular enhancement, central necrosis, peritumoral edema) does not correspond to its diffuse histopathological composition. Cell density is elevated in both CE and NE parts. Hence, our study suggests that NE contains considerable amounts of infiltrative tumor with a high cellularity which might be considered in resection planning.

## Introduction

MRI is the most important non-invasive diagnostic tool for the assessment of glioblastoma[1], the most common type of malignant brain tumor in adults [2]. T1- and T2-weighted sequences are complemented by functional MRI measurements such as diffusion- and perfusion-weighted MRI which provide additional pathophysiological information. Generally, all MRI sequences reflect physical properties of the tissue and are not necessarily tumor specific. In particular, contrast-enhancing parts on T1-weighted images reflect a disruption of the blood brain barrier that enables contrast agents to leak in the surrounding tissue and do not necessarily represent solely viable tumor tissue. [3]

To be aware of the degree of concurrence of this MRI-based classification with the actual microscopic appearance of a glioblastoma is of importance in research and clinical practice. In the former, this especially holds true for region of interest (ROI)-based analysis of different functional MRI parameters where the cT1 image is often used as a draft for the ROI.

In the latter, this might be of interest for the extent of surgical resection of glioblastoma, which has shown to be a prognostic marker for patient outcome[4–8]. Currently, the resection margins of the tumor are determined by conventional microsurgery with white light, by fluorescence guided resection with 5-aminolevulinic acid or intra-operative contrast enhanced MRI[9]. The absence of residual contrast enhancement on cT1 is usually interpreted as macroscopic complete resection. But in regard to the fast recurrence of glioblastomas after surgery even with complete resection[8], it is of interest to know to what extent the zone of T1-contrast enhancement does in fact correspond to the area of tumor infiltration and whether it is suitable to demarcate the margins of the main focus of the glioblastoma.

In this study, we correlated 561 tumor biopsies of 37 patients with newly diagnosed glioblastoma with the corresponding areas on the intraoperative contrast enhanced T1-weighted MRI to determine the accuracy of the contrast enhanced T1 MRI to distinguish necrotic foci from areas of infiltrative tumor with high cellularity.

## Materials and Methods

### Patients

This retrospective study was approved by the local ethics committee of the University of Heidelberg. Due to the retrospective nature of the study and the reduced life expectancy of the glioblastoma patients, informed consent was waived by the ethical committee. All patients had consented to the scientific use of their data with admission to our hospital. The database of our department of neuropathology was screened for patients who had undergone stereotactic biopsy between January 2010 and December 2013 and were thereafter diagnosed with glioblastoma WHO grade IV. Thirty-seven patients (18 male, 19 female, median age 63 ± 13 years) with an intraoperative MRI scan during biopsy surgery were selected. Other analyses based on the same patient cohort are reported in Eidel et al [10]

### MRI imaging

Each patient received an intraoperative MRI scan for trajectory planning which was performed in a 1.5 Tesla MRI scanner (Syngo MR B15, Siemens AG Healthcare, Erlangen) with the following parameters: TR = 9ms, TE = 2.38ms, flip angle 10°, FoV 260x260mm, voxel size (1.035mm)^3^, image matrix 256x256.

### Biopsies and trajectory planning

**Fig 1:**
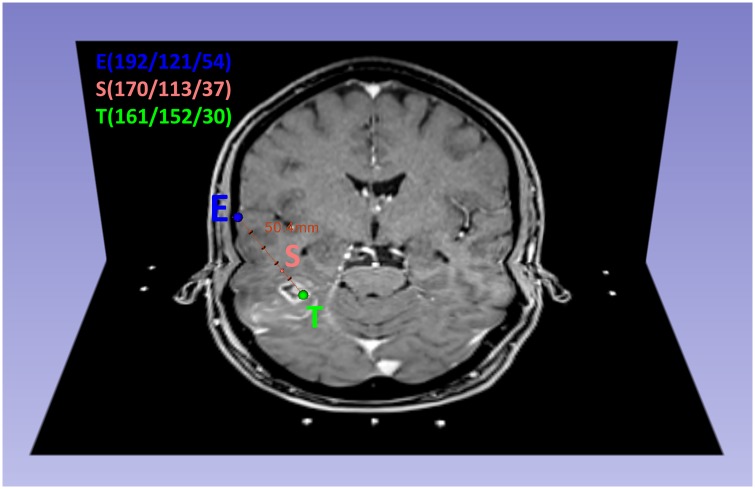
Calculation of the biopsy point S that is located in the enhancing tumor area (CE). Combination of an axial and a coronal slide of intraoperative cT1 MRI in a 60-year-old patient with glioblastoma. Cranial view. The biopsy point S is marked within the NE-area of the tumor. The trajectory (red line, length: 50.4mm) and the biopsy point S (170/113/37) were calculated via vector analysis in MATLAB from the coordinates of the known entry point E (192/121/54) and the target point T (161/152/30). The distance between S and T was 15.0 mm.

The department of neurosurgery performed the biopsy 1–21 days (median 5 days) after the first suspected diagnosis of a glioblastoma (which was based on MR imaging) and before any therapy had been administered. Under general anesthesia, a stereotactic biopsy ring was adjusted to the patient’s skull. Via intraoperative MRI, the attending neurosurgeon calculated a trajectory (iPS software, inomed Medizintechnik GmbH, Emmendingen, Germany) from an entry point at the skull to a target point in the contrast enhancing zone of the cT1 image. A total of 9–22 (median: 15) biopsies were taken along the trajectory and labeled to reconstruct their exact point of origin. The entry point and the target point of the biopsy trajectory were transferred to the intraoperative cT1 image using a custom in-house MATLAB script (MATLAB 2014b, The Mathworks, Natick, MA, USA). They formed the origin and the terminal point within a Cartesian coordinate system from which the unit vector of the trajectory could be derived via vector analysis. Thus, the exact coordinates of all biopsy points along the trajectory could be calculated as the distance between each biopsy point that was given in the pathology report (Fig 1).

We obtained a total of 561 biopsy samples, approximately 1mm^3^ in size that were sent to the department of neuropathology for further analysis and diagnosis. Each specimen was cut into 4–8 slices and stained with hematoxylin and eosin (HE stain). All biopsies were graded as glioblastoma (WHO grade IV) by a neuropathologist. For further post-processing, they were scanned at x20 magnification and saved as NDPI files.

### Postprocessing of the biopsies

**Fig 2:**
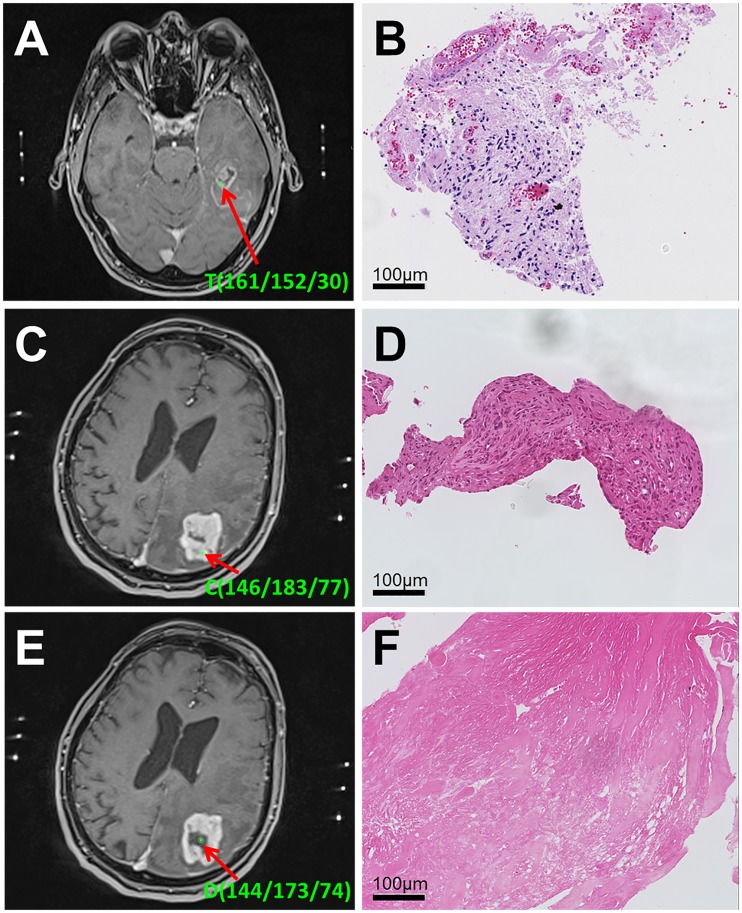
Correlation of cT1 MRI and histology. A) Target point T with its coordinates in an axial slide of the intraoperative cT1 MRI of the patient from Fig 1a. It lies in the CE area. B) Corresponding slice of the 1mm^3^ biopsy specimen (HE stain) in x20 magnification which was classified as “necrosis with cellular component”. This type of histology occurred in 31% of all biopsies originating from CE. C) Calculated biopsy point C with its coordinates in an axial slide of the intraoperative cT1 MRI of an 82-year-old patient with glioblastoma. It lies in the CE area. D) Corresponding slice of the 1mm^3^ biopsy specimen (HE stain) in x20 magnification which was classified as “viable tumor tissue”. This type of histology occurred in 60% of all biopsies originating from CE. E) Different biopsy point D with its coordinates along the trajectory in the same patient. It lies in the NEC area. F) Corresponding slice of the 1mm^3^ biopsy specimen (HE stain) in x20 magnification which was classified as “pure necrosis”. This type of histology occurred in 4% of all biopsies originating from NEC.

All biopsies were categorized into four groups by consensus of a neuropathologist (FS) and a neuroradiologist (AR) according to their histologic appearance: predominantly blood cells, pure necrosis without cells, necrosis with a cellular component (>50% necrosis) or viable tumor tissue; viable tumor tissue is defined as mostly tumor tissue (≤50% necrosis) with tumor cells. Moreover, they were assigned to 3 groups according to their position on the cT1 MRI: necrosis (NEC), contrast-enhancement (CE), or peritumoral non-enhancing (NE) (Fig 2).

**Fig 3:**
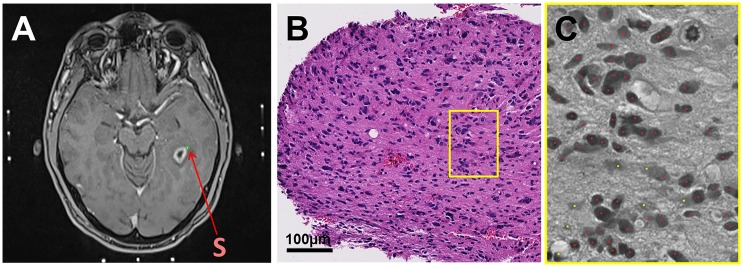
Correlation of biopsy point S with histology and semi-automatic cell counting. A) Biopsy point S is located in the NE area on an axial slide of the intraoperative cT1 MRI. B) Corresponding slice of the 1mm^3^ biopsy specimen (HE stain) in x20 magnification which was classified as “viable tumor tissue”. This type of histology occurred in 89% of all biopsies originating from NE. C) Example of semi-automatic cell counting with the ImageJ plugin ITCN. Correctly recognized tumor cells are marked red. Yellow dots are falsely detected areas of apoptotic cells or intercellular space.

Biopsy specimen histologically classified as viable tumor tissue or necrosis with cellular component were further post-processed for cell density calculation using NIH ImageJ, 64-bit version[11]. First, images were opened using NDPI Tools[12] or split into smaller-sized images if opening failed and subsequently converted to 8-bit (grey-scale). Cell density was calculated semi-automatically with the ImageJ plugin ITCN in multiple steps: First, up to 8 representative regions of cells were selected. Then, cell density calculation was performed on each region by entering an estimate of cell width and cell spacing as input. The ITCN plugin (Fig 3). A neuropathologist (FS) checked the correctness of cell detection. Cell density was then calculated for each slice per biopsy specimen and averaged.

### Statistical analysis

Statistical analysis was performed with the R language and environment for statistical computing (version 3.2.2, R Foundation for Statistical Computing, Vienna, Austria). First, the histological composition of the three different MRI categories (CE, NE and NEC) was analyzed. The percentage of biopsies classified as “necrosis” (with or without cellular component), “blood” or “viable tumor tissue” in each of the three MRI categories was calculated and compared using a χ^2^ test. Second, the cell density in the three different MRI categories was compared. Boxplots of the cell densities in the different MRI categories were created. Besides, the mean (and standard deviation) of the cell densities in the different MRI categories was calculated. Contrast analyses in a linear model and in a linear-mixed model including a random intercept for the individual patients were carried out to test whether the cell density was significantly different in biopsies from the three different MRI categories (one contrast comparing NEC and CE/NE, and another comparing CE and NE).

## Results

### Histological composition of the different MRI classifications

Of the total 561 biopsies, 321 (57.2%) originated from CE, 103 (18.4%) from NE and 137 (24.4%) from NEC. Histologically, 327 biopsies (58.3%) were classified as viable tumor cells, 175 (31.2%) as necrosis with cellular component, 11 (2.0%) as pure necrosis and 48 (8.5%) as blood cells (Table 1).

**Table 1:** Biopsy counts by histologic and MRI-based classification.

		Histologic classification			
		Viable tumor cells	Necrosis with cellular component	Pure necrosis	Blood cells
**MRI classification**	**NE**	92	5	3	3
	**CE**	194	100	3	24
	**NEC**	41	70	5	21

A χ^2^ test revealed a significant relationship between the patterns of histopathologic composition and the classification of the MRI-location in which the biopsy was found (χ^2^(6) = 92.29, p < 0.001).

**Fig 4:**
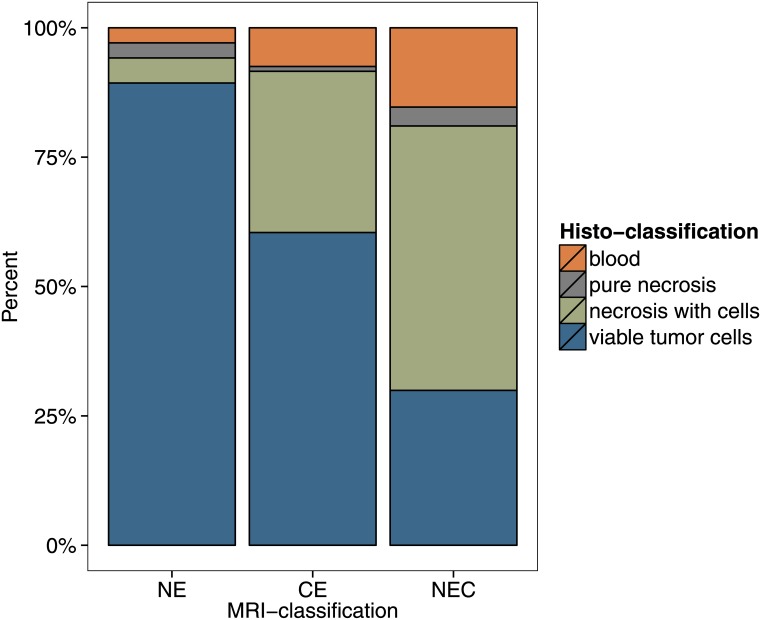
Histological composition of the different MRI classifications. For all 561 biopsy samples, the relative frequency of the different histologic classifications within each MRI classification is displayed. NE = non-enhancing part on cT1; CE = contrast enhancement on cT1; NEC = Necrosis on cT1.

The relative frequency of the different histologic classifications within each MRI classification is displayed in Fig 4. We found that areas of contrast enhancement (CE) were composed of 60% viable tumor cells, 31% necrosis with cellular component, 1% pure necrosis and 8% blood cells. Necrotic areas on the MRI (NEC) contained 30% viable tumor cells, 51% necrosis with cellular component, 4% pure necrosis and 15% blood cells. The non-enhancing part on the cT1 MRI (NE) contained 89% viable tumor cells, 5% necrosis with cellular component, 3% pure necrosis and 3% blood cells. A χ^2^ test revealed a significant relationship between the patterns of histopathologic composition and the classification of the MRI-location in which the biopsy was found (χ^2^(6) = 92.29, p < 0.001). Comparing the NE zone to the CE and NEC zone, the relative content of “viable tumor cells” was significantly higher in the NE zone (χ^2^(2) = 86.73, p < 0.001). This was also confirmed in a generalized linear model using a binomial link function (both with and without random intercept for the individual patients).

### Cellularity

**Fig 5:**
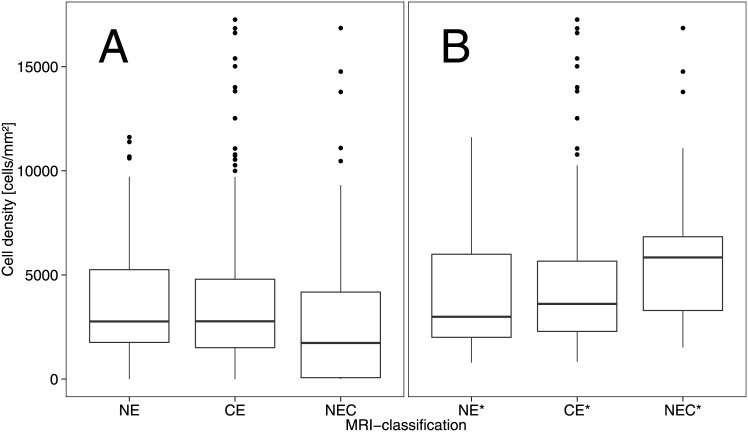
Boxplots of the cell densities in each MRI compartment. A) Cell densities for all biopsies (“viable tumor cells”, “necrosis with cellular component”, “pure necrosis”, “blood cells”). B) Cell densities in biopsies with “viable tumor cells” only. NE = non-enhancing part on cT1; CE = contrast enhancement on cT1; NEC = Necrosis on cT1.

Mean cell density within all biopsies that were classified as “viable tumor cells” was 4557 ± 3169 cells/mm^2^. In the category “necrosis with cellular component”, cell density was 2255 ± 2204 cells/mm^2^. Boxplots of the cell densities in the MRI categories are displayed in Fig 5. The mean cell densities per MRI classification are summarized in Table 2.

**Table 2:** Cell densities within MRI classifications.

MRI classification	All biopsies	Biopsies with “viable tumor cells”
**NE**	3764 ± 2893	4130 ± 2817
**CE**	3506 ± 3116	4495 ± 3209
**NEC**	2713 ± 3239	5804 ± 3480

Mean cell densities (+ standard deviation) [cells/mm^2^] within the different MRI classifications separately for all cells and for biopsies with “viable tumor cells” only.

Split up into the MRI classifications and assuming that the tumor cell density in “blood cells” and “pure necrosis” was zero, the cell density in MRI-necrosis was significantly lower than in the other two MRI classifications in the linear model (t(558) = -2.90, p = 0.004)(Table 2 and Fig 5A). Moreover, cell densities did not differ significantly between CE and NE (t(558) = -0.73, p = 0.46). Repeating the analyses with a linear-mixed model, both comparisons were not significant (t(557.8) = -0.65, p = 0.52 for NEC vs. CE/NE, and t(557.7) = 1.59, p = 0.11 for CE vs. NE).

If we only consider cell density within the biopsies that classified as “viable tumor cells”, excluding necrotic parts and bleeds that dilute overall cell density, cell density was highest in NEC* compared to the other two MRI classifications (t(324) = 2.82, p = 0.005) (Table 2 and Fig 5B). Additionally, CE* showed a higher cell density than NE*–however, this difference was not significant (t(324) = 0.92, p = 0.36). In a linear-mixed model, both comparisons reached statistical significance (t(319.1) = 4.29, p < 0.001 for NEC* vs. CE*/NE*, and t(319.6) = 2.90, p = 0.004 for CE* vs. NE*).

To account for potential non-normality in the residuals of the dependent variable, the analyses were repeated using non-parametric Mann-Whitney-U tests which replicated the results. In addition, using the square root transformed cell density as dependent variable in the linear(-mixed) models also led to comparable results—with the exception that the difference between NEC and CE/NE for all cells was now also significant in the linear-mixed model.

## Discussion

This study illustrates that the appearance of a glioblastoma on a cT1 image (circular enhancement, central necrosis, peritumoral edema) does not strictly correspond to its diffuse histopathological composition. NE had the highest relative content of viable tumor cells and an average cellularity comparably high to CE which suggests that considerable infiltration or even the main tumor burden occur beyond the contrast enhancing part.

If we only take into account biopsies that were classified as “viable tumor cells”, highest cell density is found in the central parts of the tumor (“MRI necrosis”) and decreases significantly towards the periphery. It is well established that glioblastomas are disseminated tumors with a fringe of invasive cells around a core lesion[13] and they have often spread throughout the brain at initial diagnosis[14, 15]. Still, maximum safe resection of the visible lesion is important[4–8], particularly because rapidly migrating cells are thought not to proliferate whereas proliferating cells tend not to migrate and recurrences usually occur near the area of initial resection[13].

Other studies are in accordance with our findings, although the proposed problem and the methodic approach were different.

Kubben et al[16] examined 10 patients (36 biopsy samples) with glioblastoma in an intraoperative contrast enhanced 0.15 Tesla MRI after the first resection of the tumor and obtained neuronavigation-guided biopsies at the border of the resection cavity together with a screen capture to relate contrast enhancement with histopathology. Other than stating that contrast enhancement after excision is due to residual tumor rather than iatrogenic tissue manipulation, they found a correlation between the presence of contrast enhancement and increased cellularity (Kendall’s tau T_k_ = 0.26), necrosis (T_k_ = 0.49), vascular changes (T_k_ = 0.53) and WHO grade (T_k_ = 0.5) in the biopsy sample, but did not compare it to values from NE regions. The resolution of the 0.15 Tesla scanner that Kubben et al used is very low, which is also a limitation of the study of Kelly et al[17] on 40 patients with brain tumors of different entities. 195 biopsy samples were compared to CT and unenhanced 0.15 Tesla MRI images. They found that tumor cell infiltration extended at least as far as was indicated by the prolongation of T2 on the MRI but were unable to differentiate edematous from infiltrated parenchyma according to the prolongation of T1 and T2.

There are three studies that state a higher cellularity in CE compared to NE.

In an analysis of 30 samples (13 patients) from preplanned 75mm^2^ region of interests, Barajas et al[18] found that CE biopsy sites had significantly elevated overall cellularity compared with peritumoral NE biopsy regions (p < 0.01). This is a result of manual quantification of the biopsies as the average of the total number of cells within three high power fields at a magnification of x20 (1.0 mm^2^).

In a more recent study[19], the same research group took biopsies during glioblastoma resection on preselected sites within or outside the contrast enhancement on the preoperative MRI. They biopsied if the Apparent Diffusion Coefficient (ADC) value < 1200, relative peak height from DSC MRI > 3 or choline to N-acetyl aspartate index (CNI) > 2 SEs above normal. Tissue specimens obtained from CE regions had significantly higher tumor cell density compared to NE regions (tumor cell density mean 268 vs. 146 total cells per field at x200 magnification, p = 0.007).

Gill et al[20] performed an analysis of MRI-localized biopsies of 69 glioblastoma patients with an emphasis on the composite gene expression profile within enhancing and non-enhancing parts of a glioblastoma. They also found a significantly higher cellularity in samples from CE compared to NE regions.

Our results on the other hand suggest that the cell density within CE and NE is generally comparable (however, there was a statistically significant difference if only biopsies with entirely tumorous appearance were considered in a linear-mixed model). Interestingly, the proportion of “viable tumor cells” in NE in our study is higher than in CE, which is interspersed with necrosis. This indicates that the main burden of proliferative tumor cells is located in NE. Hence, adding a qualitative category to the analysis of the biopsies instead of merely counting the cells provides a better understanding of what the glioblastoma must look like in the different MRI locations. In this regard, necrosis was present in 40% of CE samples in the study of Barajas et al, which is in accordance with our results.

The stated discrepancy in regard to the cell densities in CE and NE might be explained as all three studies performed a manual cell count that does not achieve the same accuracy as semi-automatic cell counting. As only distinct high-power-fields are chosen for counting, the approach is also observer-dependent. The only limitation of semi-automatic cell counting on the other hand is that they do not account for signs of malignancy in cells such as cytologic atypia, enlarged nuclear to cytoplasmic volume ratio or hyperchromasia. The algorithm was trained to detect tumorous cells and in most cases was able to distinguish these from benign or apoptotic brain cells due to slight differences in cell width and spacing. However, it cannot be guaranteed that some of the latter cells have been falsely detected. As only HE stains were available, differentiation of aforementioned histological features was limited. Furthermore, the decision to apply categories to the histologic classification might narrow the content of information that can be obtained from the biopsies, but it allows us to focus on distinguishing the infiltrative tumor parts (“viable tumor cells”) from the rest of the tumor mass (“blood cells”, “pure necrosis”, “necrosis with cellular component”). With every classification, there is also a certain observer dependency, which we tried to minimize by consensus reading of a neuroradiologist and a neuropathologist.

In regard to the studies of Barajas et al. [18, 19], the pre-selection of biopsy sites by their functional MR values (ADC, Cerebral Blood Volume (CBV)) may have been a possible confounder, as the ADC itself is reported to be inversely correlated with cellularity [21] and CBV might show a trend to a positive correlation [22].

Another limitation of our study is that NE and CE biopsy regions were in nearly all cases adjacent. This technical limitation which is caused by the direction of the stereotactic biopsy trajectory might constitute a further element of bias.

A further limitation of this study is that exclusively T1-weighted intraoperative images were included, as it is well known that T2-hyperintense areas include invasive parts of glioblastoma [23, 24]. However, T2-hyperintensities may also be caused by multiple confounding conditions (e.g. edema, demyelination, ischemic injury, seizures) [3]. Hence, in future studies it may be of interest to perform the histopathological correlational analysis on the basis of T2-weighted images.

The strengths of our study are the high resolution of the 1.5 Tesla MRI and the large sample of 561 biopsies, exclusively obtained from glioblastoma patients. Also, the allocation of the biopsies to the MRI is precise and observer-independent in our approach.

From a clinical point of view, our study might advocate larger margins for resection or radiation therapy for patients with newly diagnosed glioblastoma. However, no firm conclusions can be drawn from the current correlational study and future carefully designed studies—outweighing risk and benefit for the patient—might address such an approach.

In conclusion, contrast enhanced T1-weighted MRI cannot reliably distinguish necrotic foci from areas of infiltrative tumor with high cellularity. The non-enhancing part on the cT1 MRI (NE) seems to correspond best to the zone of tumor infiltration and contains a high tumor burden which is why a resection margin that exceeds the widely used contrast enhancement on cT1 has to be considered in the excision of glioblastoma. This finding is also important for studies that correlate MRI parameters with tumor biology on a region of interest-based approach.
